# The senescent bystander effect is caused by ROS-activated NF-κB signalling

**DOI:** 10.1016/j.mad.2017.08.005

**Published:** 2018-03

**Authors:** Glyn Nelson, Olena Kucheryavenko, James Wordsworth, Thomas von Zglinicki

**Affiliations:** The Ageing Biology Centre, Campus for Ageing and Vitality, Newcastle University, Newcastle upon Tyne NE4 5PL, UK

**Keywords:** Senescence, Mitochondria, Reactive oxygen, NF-κB, Bystander

## Abstract

Cell senescence is an important driver of the ageing process. The accumulation of senescent cells in tissues is accelerated by stress signals from senescent cells that induce DNA damage and ultimately senescence in bystander cells. We examine here the interplay of senescence-associated mitochondrial dysfunction (SAMD)—driven production of reactive oxygen species (ROS) and senescence-associated secretory phenotype (SASP) in causing the bystander effect. We show that in various modes of fibroblast senescence ROS are necessary and sufficient to activate the transcription factor nuclear factor kappa-light-chain-enhancer of activated B cells (NF-κB), which facilitates a large part of the SASP. This ROS-NF-κB axis causes the DNA damage response in bystander cells. Cytokines IL-6 and IL-8 are major components of the pro-inflammatory SASP in senescent fibroblasts. However, their activation in senescence is only partially controlled by NF-κB, and they are thus not strong candidates as intercellular mediators of the bystander effect as mediated by the ROS-NF-κB axis.

## Highlights

• Mitochondrial dysfunction in cell senescence is sufficient for NF-κB activation via ROS.
• NF-κB activation drives the senescent bystander effect.
• IL-6 and IL-8 secretion in senescence is only partly dependent on NF-κB.
• IL-6 and IL-8 are not essential mediators of the bystander effect.

## Graphical abstract

**Figure:**
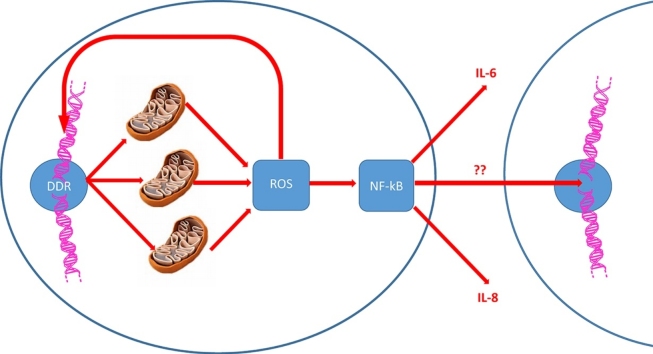

## 1 Introduction

It is becoming increasingly clear that cell senescence is an important cause of the loss of tissue functionality and homeostatic capacity that characterizes ageing. Senescent cells accumulate in many, if not all, tissues with age (Dimri et al., 1995, Herbig et al., 2006, Jurk et al., 2012, Wang et al., 2009). In mice, the rate of this accumulation predicts mean and maximum lifespan of animals over an almost threefold difference in life expectancy between slow- and fast-ageing cohorts (Jurk et al., 2014). Selective ablation of senescent cells postponed the development of age-associated tissue dysfunction/disease and extended mean lifespan in mice (Baker et al., 2016, Baker et al., 2011). Drugs like rapamycin, which extend life expectancy in mice, have been shown to be senostatics, i.e. suppressors of multiple aspects of the senescent phenotype (Correia-Melo et al., 2016). First experimental drugs that selectively kill senescent cells have been developed and show encouraging results in a number of mouse models of age-related diseases (Roos et al., 2016, Schafer et al., 2017, Zhu et al., 2015).

An important role of cell senescence in ageing should not come as a surprise. After all, cell senescence is a major response to stress and molecular damage. While it was originally regarded only as a consequence of ongoing cell division and associated telomere loss, seminal work from Toussaint and others soon showed that both telomere-dependent and independent senescence can be induced by DNA-damaging stress (Toussaint et al., 2000, von Zglinicki et al., 1995) acting via the by now well-defined DNA damage response (DDR) pathway (d’Adda di Fagagna et al., 2003). As was predicted early (von Zglinicki et al., 2005), the distinction between telomere-dependent (replicative) and telomere-independent stress-induced senescence became more and more blurred with data showing stress-related telomere loss as predictor of transition to senescence at the single cell level (Passos et al., 2007) as well as the defining role for telomere damage without telomere loss for senescence in mouse cells in vitro and in vivo (Hewitt et al., 2012, Jurk et al., 2014). Moreover, cell senescence is also the response to stresses that are not primarily genotoxic but impact preferentially on the proteome (Castro et al., 2012) or the mitochondrion (Miwa et al., 2014, Wiley et al., 2016).

In addition to being a stress response, cell senescence is itself an inducer of stress in the tissues and organisms harbouring senescent cells. This is because of three important properties of senescent cells: i) Senescent cells cannot proliferate. Accordingly, senescence of stem and progenitor cells limits the regenerative potential of tissues and organisms. ii) Senescent cells hyper-produce and secrete a host of bioactive peptides (the so-called Senescence-Associated Secretory Phenotype SASP), prominently including pro-inflammatory cytokines like IL-1, IL-6 and IL-8 under control of the transcription factor NF-κB (Coppe et al., 2008). These not only stabilize senescence in an autocrine fashion (Acosta et al., 2008, Kuilman et al., 2008) but may also be a significant cause of age-related chronic sterile inflammation and contribute to bystander effects, i.e. enhanced tumorigenicity of transformed cells (Krtolica et al., 2001) and induction of DNA damage and senescence in normal bystander cells (Nelson et al., 2012). iii) The activated DDR in senescent cells also causes Senescence-Associated Mitochondrial Dysfunction (SAMD), i.e. loss of mitochondrial membrane potential, decreased respiratory coupling and increased ROS production, all associated with failing mitophagy (Dalle Pezze et al., 2014, Korolchuk et al., 2017, Passos et al., 2010). Importantly, if mitochondria were selectively and specifically obliterated in senescent cells, ROS levels returned to normal, indicating SAMD rather than extra-mitochondrial mechanisms as the major source of elevated cellular ROS in senescence (Correia-Melo et al., 2016). As for SASP, SAMD initiates an autocrine positive feedback loop that enhances ROS-mediated nuclear DNA damage and thus stabilizes senescence (Passos et al., 2010). In its turn, ROS release from senescent cells contributes to senescence induction in bystander cells (Nelson et al., 2012). Finally, SAMD appears as a cause for metabolic insufficiencies in ageing organisms (Ogrodnik et al., 2017).

To understand better how senescent cells contribute to ageing, it will be necessary to dissect how these two major senescent phenotypes, SAMD and SASP, interact. It is clear that the two can influence each other: Short telomeres do not only cause senescence in late generation telomerase knockout mice (Choudhury et al., 2007), they also induce widespread inflammation (Jurk et al., 2014). Conversely, chronic inflammation in mice null for the repressor p50 subunit of NF-κB aggravates ROS production and senescence (Jurk et al., 2014).

To further assess the interaction between SASP and SAMD, we treated human fibroblasts in various modes of senescence with either antioxidants or NF-κB inhibitors and monitored the effects on the corresponding phenotype. We also analyzed the relative efficiencies to induce a DDR in bystander cells under SASP- vs SAMD-inhibition. We found that antioxidants suppress activation of NF-κB, while suppression of NF-κB did not reduce SAMD in multiple senescence modes. Both scavenging of ROS and inhibition of NF-κB blocked the bystander effect. We conclude that SAMD is an important inducer of the pro-inflammatory SASP, which then mediates the bystander effect.

## 2 Results

### 2.1 SAMD-derived ROS control SASP but SASP does not control SAMD

**Fig. 1:**
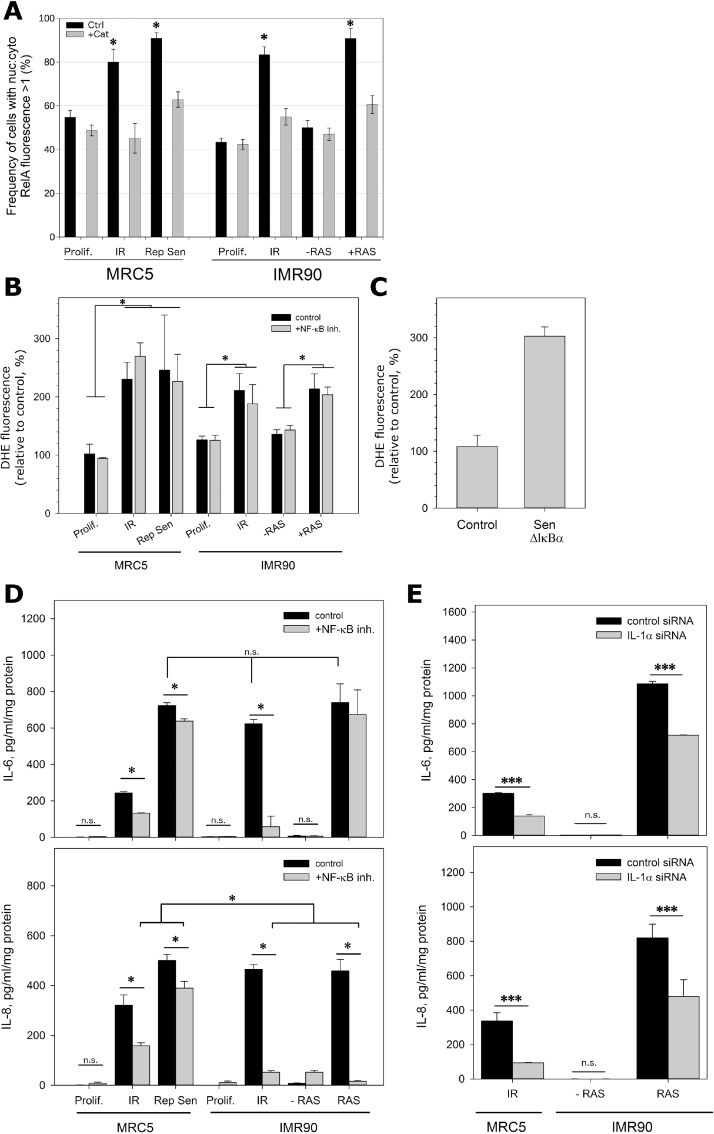
**ROS controls SASP, but SASP does not control SAMD. A: ROS release from senescent cells is necessary for activation of NF-κB**. Nuclear and cytosolic localization of the NF-κB transcription factor, RelA, was measured at single cell level by immunofluorescence. Frequencies of cells with nuclear: cytosolic NF-κB signal ratio >1 are shown. Cells were untreated (Ctrl) or treated with catalase (100 U/ml for 2 days prior to fixation, +Cat). Experiments were performed three times, data shown are means +/− SD. Statistical tests were performed as one way ANOVA with a Dunn's post-hoc test. **B: NF-κB activity does not impact on senescence-associated ROS generation**. Cultured cells were grown with and without NF-κB inhibitor (Bay11-7082, 1 μM) for 2 days prior to measuring DHE fluorescence by flow cytometry. All modes of senescence increased fluorescence intensity, but there were no differences between cells treated with the NF-κB inhibitor compared to their respective controls. Each experiment was performed three times, data shown are means +/− SD. Statistical tests were performed as one way ANOVA with a Dunn's post-hoc test. **C: Transgenic inhibition of NF-κB signalling does not affect ROS production in replicative senescence**. MRC5 transduced with a lentiviral dominant negative IκBα −IRES-EGFP construct were analysed as actively proliferating as well as grown to replicative senescence for DHE fluorescence by flow cytometry. Each experiment was performed three times, data shown are means +/− SD. Statistical tests were performed as a Student's T-test (proliferating control versus Rep Sen), and also as a one way ANOVA with parental MRC5 cells (shown in B), which showed no significant difference between the two replicatively senescent populations. **D: SASP cytokines are partially controlled by NF-κB in replicative and stress-induced senescence**. ELISA measurements of IL-6 (top) and IL-8 (bottom) in medium from cells as indicated treated with and without NF-κB inhibitor (Bay11-7082, 1 μM). Experiments were performed twice (duplicate cultures ran in triplicate), data plotted as mean +/− SD. Statistical tests were performed as two way ANOVA with Holm-Sidak post-hoc tests. **E: IL-1α controls SASP cytokines in two senescence modes**. ELISA measurements of IL-6 (top) and IL-8 (bottom) in medium of cells 3 days after transfection with IL-1α siRNA or scrambled control siRNA. Experiments were performed twice (duplicate cultures ran in triplicate), data plotted as mean +/− SD. Statistical tests were performed using a one way ANOVA with Holm-Sidak post-hoc tests.

There has been sometimes contradictory discussion about the impact of factors such as mode of senescence induction, cell strain, mitochondrial (dys-)function, etc. on SASP composition and potential downstream bystander effects. We therefore tested the interaction between SASP and SAMD in multiple human fibroblast systems, using two independent cell strains (MRC5 and IMR90) and three different modes of senescence. Cellular senescence was induced via either proliferative exhaustion (replicative senescence in MRC5, ‘RS'), IR stress-induced senescence (20 Gy X-ray irradiation in MRC5 and IMR90, ‘IR') or oncogene induced senescence (Ras induction in a stably transfected IMR90 cell line, ' + RAS'). Successful induction of senescence was confirmed by reduction of growth to less than 10% of controls, senescent morphology and Sen-β-Gal positivity in at least 80% of the cells (data not shown). NF-κB activity was measured as the relative nuclear/cytoplasmic intensity of the immunofluorescence signal from the predominant NF-κB transcription factor subunit RelA (Suppl. Fig. S1A) (Gilmore, 2006). The assay was standardized by showing an increase in nuclear RelA fluorescence following stimulation of NF-κB with 10 ng/ml TNFα (Suppl. Fig. S1B). Fibroblasts in all senescence modes displayed significantly higher levels of nuclear RelA than the proliferating control cells (Fig. 1A). Cellular ROS levels were measured by DHE fluorescence. While we are aware that DHE fluorescence intensity is a marker but not a quantitative measure of cytoplasmic superoxide, and that cytoplasmic ROS originate from multiple sources including non-mitochondrial ones, we have previously shown that the increase in ROS markers associated with senescence can solely be attributed to mitochondria (Correia-Melo et al., 2016). Therefore, we regard the increase of DHE fluorescence above basal levels in senescent cells as a simple indicator of SAMD. Accordingly, all senescent fibroblasts displayed increased DHE fluorescence (Fig. 1B) in accordance with earlier data (Dalle Pezze et al., 2014, Passos et al., 2010, Passos et al., 2007). Treatment of senescent cells for 2 days with the antioxidant catalase (CAT) depleted intracellular ROS levels in senescent cells, but had little effect on the low ROS levels in proliferating cells (Suppl. Fig. S2). Importantly, catalase treatment completely rescued the senescence-associated activation of NF-κB (Fig. 1A), suggesting that SAMD-mediated ROS production is necessary and sufficient for NF-κB activation in senescence. In contrast, inhibition of NF-κB with the specific inhibitor, Bay 11-7082 (1 μM for 2 days) reduced NF-κB activation in senescent cells as expected (Suppl. Fig. S3), but had no effect on ROS levels (Fig. 1B), indicating that the SAMD-mediated ROS hyper-production was independent from NF-κB signaling in all senescence modes. To corroborate this finding, we stably over-expressed a dominant negative NF-κB suppressor, ΔIκBα (Heimberg et al., 2001) in MRC5 fibroblasts and grew them to replicative senescence. While this was effective in suppression of TNFα-mediated NF-κB activation (Suppl. Fig. S1B), it again did not diminish ROS production in senescent cells (Fig. 1C).

IL-6 and IL-8 are two important pro-inflammatory cytokines and major components of the SASP in human fibroblasts. They are under control of NF-κB, mediated partly via induction of IL-1 (Coppe et al., 2008). However, other transcription factors, notably C/EBPβ, also determine expression of these cytokines in senescence (Kuilman et al., 2008). Thus, ROS might stimulate the SASP independently of NF-κB activity. Therefore, we next assessed how NF-κB, IL- 1α and ROS determined the amount of IL-6 and IL-8 secreted from senescent cells. As expected, secretion of both IL-6 and IL-8 was enhanced in all modes of fibroblast senescence (Fig. 1D). Interestingly, inhibition of NF-κB had differential effects depending on senescence mode and interleukin species: It was highly effective for both IL-6 and IL-8 in IR-induced senescence (slightly less so for MRC5 than for IMR90) but resulted only in a minor inhibition of secretion of both peptides in MRC5 replicative senescence. In IMR90 + RAS, it was highly effective for IL-8 but did not change IL-6 secretion at all (Fig. 1D). These data indicate variable roles for signaling from the transcription factor NF-κB in SASP, depending on senescence model and specific interleukin. To test whether this variation might arise at the level of IL-1α expression, we knocked down IL-1α in two senescence models, MRC5-IR and IMR90 + RAS, which differed in their IL-6 and IL-8 response to inhibition of NF-κB (Fig. 1D). Transfection with anti-IL1α siRNA reduced its mRNA (Suppl. Fig. S4A) and protein levels (Suppl. Fig. S4B) similarly in both senescence modes. It also impacted equally on IL-6 and IL-8 release in both forms of senescence (Fig. 1E), suggesting that the different responses of these cytokines to NF-κB inhibition are not mediated at or downstream of cytokine IL-1α expression.

### 2.2 SAMD −induced SASP causes the senescent bystander effect

**Fig. 2:**
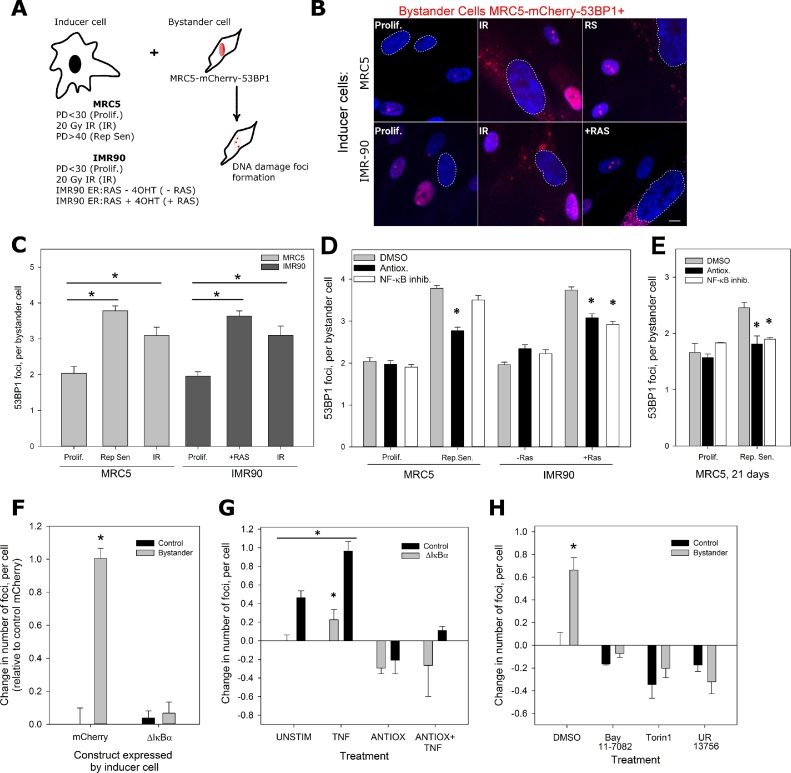
Role of SASP and SAMD for the bystander effect. **A: Cartoon model representing the in vitro model system designed to analyse the senescent bystander effect**. Three different modes of cellular senescence were employed in the inducer cells: replicative senescence in MRC5 (Rep Sen), oncogene induced senescence via RAS in IMR90 (OIS), and X-ray irradiation induced senescence (10 days post 20 Gy) of either MRC5 or IMR90 (IR). In each case, young proliferating MRC5 cells stably expressing mcherry-53BP1 were used as bystander cells, cultured in a 1:1 ratio, and mcherry-53BP1 foci were counted in bystander cells to determine the level of DNA damage. **B: Representative images of co-cultured bystander cells** (mCherry-53BP1 expressing MRC5) in the presence of inducer cells (MRC5 top, IMR90 bottom). The mode of senescence for each inducer type is shown in the image overlays. Dashed outlines highlight inducer cell nuclei. Blue represents DAPI staining, red mCherry-53BP1. Note cytosolic autofluorescence present in senescent inducers. Scale bar = 10 μm. **C: All modes of senescence induce a bystander response**. Co-cultures were grown for 7 days with either young proliferating control cells (Prolif.) or inducer cells in the indicated senescence mode. 53BP1 foci frequencies in the bystander cells are shown. Significant differences (* P < 0.05) in comparison to respective controls are indicated, Kruskal-Wallis ANOVA on Ranks with Holm-Sidak post hoc test for pairwise comparison between groups. Neither cell strain nor senescence mode had a differential impact on the bystander effect (Two-way ANOVA test). Data plotted are mean +/− SD from 100 to 150 cells per group from 3 independent replicates. **D, E: ROS and NF-κB contribute to the bystander response.** Proliferating control (Prolif or −RAS) or senescent inducer cells as indicated were co-cultured with proliferating MRC5:53BP1 bystander cells in the presence of either Catalase (Antiox., 100U/ml), NF-κB inhibitor Bay11-7082 (1 μM) or carrier (DMSO). Cultures were co-cultured for either 6 days (**D**) or 21 days (**E**). 53BP1 foci frequencies in the bystander cells are shown. CAT significantly inhibited the bystander effect in all experiments, while Bay11-7082 reduced the bystander effect from MRC5-RS only after prolonged co-culture. Data plotted are mean +/− SD from 180 to 250 cells per group from 2 independent replicates. Kruskal-Wallis ANOVA on Ranks with Holm-Sidak post hoc test for pairwise comparison between groups, asterisks show P < 0.05. **F: ΔIκBα expression inhibits the bystander effect from replicatively senescent MRC5**. Young (Prolif) or replicatively senescent (RS) MRC5 fibroblast stably transduced with either mCherry or ΔIκBα we co-cultured with MRC5:53BP1 bystander cells. Differences between 53BP1 foci frequencies in bystander cells to those in proliferating cells alone are shown. Experiments were performed in triplicate and between 60 and 120 cells were analysed per treatment and replicate. Data are mean ± SE (n = 3-5), with approximately 100 cells per experiment. **G: TNFα pre-stimulation enhances the bystander effect, and CAT abrogates it**. Senescent inducer cells were stimulated with TNFα (TNF) or not (UNSTIM) before co-culture with reporter cells for 21 days. CAT (ANTIOX) was added during co-culture. Differences between 53BP1 foci frequencies in bystander cells to those in proliferating cells alone are shown. Data are mean ± SE (n = 3-5), with approximately 100 cells per experiment. **H: Inhibitors of NF-κB, mTORC1 and p38MAPK equally suppress the bystander effect.** Replicatively senescent MRC5 fibroblasts were co-cultured with reporter cells for 21 days with either DMSO as carrier, Bay11-7082 (BAY), the mTORC1 inhibitor torin1 (TOR) or the p38MAPK inhibitor UR-13756. Differences between 53BP1 foci frequencies in bystander cells to those in proliferating cells alone are shown. Data are mean ± SE (n = 3-5), with approximately 100 cells per experiment. (For interpretation of the references to colour in this figure legend, the reader is referred to the web version of this article.)

In co-culture with young, proliferation-competent fibroblasts, senescent cells induce DNA damage that ultimately causes senescence in the bystander cells (Nelson et al., 2012). To investigate the contributions of SASP and SAMD to the bystander effect, we established 2D co-cultures of young MRC5 human diploid fibroblasts (bystanders) with inducer fibroblasts rendered senescent in different modes (Fig. 2A). Bystander cells were discriminated by expression of an mCherry-tagged 53BP1 which in young cells appear as diffuse nuclear red fluorescence with only occasional bright foci, while it forms intense nuclear foci upon induction of DNA damage (Nelson et al., 2009) and in senescence (Passos et al., 2010). Already after 2 days of co-culture, ROS levels in young bystander cells were enhanced and stayed above control levels for the duration of the experiment, with levels comparable to senescent cells (Suppl. Fig. S5). After 6 days of co-culture with inducer cells in any senescence mode, young bystander cells displayed increased DDR (Fig. 2B). Average frequencies of 53BP1 foci were significantly increased after co-culture with senescent, but not non-senescent inducers (Fig. 2C), irrespective of the cell strain or the senescence mode (Fig. 2C). A two way ANOVA with post hoc analysis indicated that there was no significant difference between treatment cell line, with all senescent inducers producing a significant increase in DDR compared to their young inducer controls (either MRC5 or IMR90).

We next performed the co-culture experiments with MRC5-RS or IMR90 + RAS as inducers in the presence of either the antioxidant catalase or the NF-κB inhibitor Bay11-7082. After 6 days of co-culture, the bystander effect from both types of senescence inducers was reduced by catalase, while the NF-κB inhibitor was able to suppress the bystander effect of IMR90 + RAS, but not that of replicatively senescent MRC5 inducers (Fig. 2D). This may be because Bay11-7082 suppresses IL-6 and IL-8 secretion from replicatively senescent MRC5 fibroblasts only weakly (see Fig. 1C). However, when the incubation of bystander cells with replicatively senescent MRC5 fibroblasts was prolonged to 21 days, NF-κB inhibition became as efficient in suppression of the bystander effect as incubation with catalase (Fig. 2E).

The long-term bystander effect from MRC5-RS fibroblasts was equally inhibited by expressing the NF-κB super-repressor ΔIκBα in the inducer cells only (Fig. 2F). Conversely, pre-treatment of senescent inducer cells with TNFα before co-culture enhanced the bystander effect from wild-type, but not ΔIκBα − expressing inducers (Fig. 2G). Catalase treatment was sufficient to rescue even this enhanced bystander effect (Fig. 2G).

Insufficient mitophagy is an important cause of mitochondrial dysfunction and ROS production in senescence, and activation of mitophagy by treatment with rapamycin improves mitochondrial function and suppresses the pro-inflammatory SASP (Correia-Melo et al., 2016, Dalle Pezze et al., 2014, Miwa et al., 2014). Accordingly, treatment of co-cultured inducer and bystander cells with the specific mTORC1 inhibitor Torin1 was as efficient to suppress the bystander effect as treatment with the NF-κB inhibitor Bay 11–7082 (Fig. 2H). The stress response kinase p38/MAPK14 is a major intermediate for ROS-mediated upregulation of NF-κB (Nakajima and Kitamura, 2013), and its inhibition similarly suppressed the bystander effect (Fig. 2H).

Together these data suggest that in senescence, mitochondrial dysfunction induces ROS, which activate NF-κB via p38 and possibly other pathways. This is responsible for the induction of a pro-inflammatory SASP, albeit to different degrees in different cell types and/or modes of senescence induction. SASP factors appear to be the main mediators of cell-to-cell communication, transmitting the bystander effect.

## 3 Discussion

A recent series of intervention experiments has clearly pinpointed senescent cells as an important cause of age-associated tissue dysfunction, (multi-)morbidity and, potentially, mortality (Baker et al., 2016, Jurk et al., 2014, Ogrodnik et al., 2017, Roos et al., 2016, Schafer et al., 2017, Zhu et al., 2015). Senescent cells impact on their tissue environment by a host of pathways: they secrete bioactive, especially pro-inflammatory and matrix-degrading, peptides (the SASP, (Coppe et al., 2008)), produce and release ROS as consequence of SAMD (Passos et al., 2010, Passos et al., 2007), shed microvesicles that contain (relatively) specific miRNAs (Weilner et al., 2016) and proteins (Effenberger et al., 2014) and release modified lipids (Ni et al., 2016). These may act as signaling molecules that spread senescence to bystander cells (Acosta et al., 2013, Nelson et al., 2012), thus aggravating the pro-ageing effects of senescent cells. While previous data are in agreement with the idea that SASP factors might play a major role in induction of the senescent bystander effect, other mediators have not been ruled out. In fact, effects from conditioned medium were sometimes weak in comparison to cell–cell contact or ROS-mediated effects (Nelson et al., 2012). Moreover, specific roles for individual SASP factors in transmission of the bystander effect are not known.

To understand the interactions between SAMD and SASP in the induction of bystander senescence, we examined the effects of scavenging SAMD-dependent ROS or inhibiting the major SASP transcription factor NF-κB in multiple modes of fibroblast senescence. It is known that ROS can, in a context-depending manner, either activate or inhibit NF-κB (Nakajima and Kitamura, 2013). Our results show that hyper-production of ROS in senescence is driving NF-κB activation in all examined modes of senescence. Conversely, inhibition of NF-κB did not change ROS hyper-production in senescence. This was unexpected as earlier work showed that activation of NF-κB signaling by knockout of the NFKB1 gene resulted in accelerated senescence and aggravated ROS production in senescent cells (Jurk et al., 2014). The probable explanation for this discrepancy lies in the existence of multiple pathways leading to ROS production in senescent cells: the DNA Damage Response drives SAMD and thus increases ROS to a level that is necessary to establish senescence (Passos et al., 2010); activation of NF-κB increases ROS further by pathway(s) that involve cyclooxygenase activation among others, but not enhancement of mitochondrial ROS production (Jurk et al., 2014). Thus, SAMD activates NF-κB via ROS but is not targeted by NF-κB activity, although NF-κB activation contributes to non-mitochondrial ROS production. This interpretation is supported by data showing that the pro-inflammatory SASP is suppressed in those modes of senescence where mitochondrial dysfunction does not lead to enhanced cytoplasmic (and presumably nuclear) ROS levels (Wiley et al., 2016). In phagocytes, mitochondrial ROS production and NF-κB activation are interconnected by formation of a complex between mitochondrial complex I–evolutionarily conserved signaling intermediate in Toll pathways (ECSIT) and the ubiquitin ligase tumor necrosis factor receptor-associated factor 6 (TRAF6) (Min et al., 2017, West et al., 2011). Whether this complex is also activated in fibroblasts senescence is not clear yet.

NF-κB is not the only transcription factor driving the pro-inflammatory arm of the SASP (Acosta et al., 2008). Our data show that its impact on secretion of the two major pro-inflammatory cytokines, IL-6 and IL-8, significantly depends on cell type, senescence mode and specific cytokine. In MRC5 replicative senescence, for instance, the impact of NF-κB signaling on IL-6 and IL-8 secretion was significant but rather small (Fig. 1D). In contrast, a number of interventions were all similarly effective in blocking the senescence-induced induction of a DDR in bystander MRC5 fibroblasts. Although the antioxidant reduced the bystander response earlier than the NF-κB inhibitor, both were able to rescue the bystander effect completely after prolonged incubation, and this was not different from inhibition of signaling through mTORC1 or p38MAPK. We interpret these results as follows:

ROS hyper-production in senescence is at least partially due, or aggravated by, accumulation of dysfunctional mitochondria, which in turn is mediated by a failure of mitophagy (Dalle Pezze et al., 2014, Korolchuk et al., 2017). SAMD can be rescued by inhibition of mTORC1 signalling using rapamycin (Correia-Melo et al., 2016) or torin (Dalle Pezze et al., 2014), activating autophagy. ROS signal through p38MAPK to activate NF-κB in senescent cells, and NF-κB in turn is responsible for some, but not all of the SASP. The fact that, given sufficiently long incubation, inhibitors for all tested components of this pathway effectively and completely rescued the bystander response suggest that peptides transcribed under control of NF-κB are the intercellular mediators of the senescent bystander response. However, neither IL-6 nor IL-8 seem to play major roles in this respect, as they are only to a small degree controlled by NF-κB in senescent MRC5 fibroblasts. Further work will be necessary to identify more potent intercellular signal transducer candidates for the senescent bystander effect.

## 4 Materials and methods

### 4.1 Cell culture

Proliferating cells. Embryonic human lung fibroblasts MRC5 were from ECACC. MRC5-mCherry-53BP1 cells were created as described previously (Hewitt et al., 2016). Cells were cultured in DMEM (Sigma Aldrich) supplemented with 10% foetal calf serum (Sigma Aldrich), 100 U/ml penicillin, 100 mg/ml streptomycin and 2 mM l-Glutamine at 20% oxygen, 5% CO_2_ at 37 °C. Embryonic lung fibroblast IMR90 were cultured in phenol-free DMEM with supplements as above at 3% oxygen, 5% CO_2_, 37 °C. Stably transfected IMR90ER:RAS (a gift from Peter Adams) were cultured in the same manner as the parental IMR90 plus 300 μg/ml G418 selection at 3% oxygen, sub-cultured every third day.

The NF-κB super-repressor vector pLenti-ΔIκBα-IRES-EGFP was created by first cloning IκΒα minus the first 40 amino acids from pIκB-EGFP (Clontech), into the MCS of pIRES2-EGFP (Clontech) and then the entire ΔIκBα-IRES-EGFP cassette into the expression site of pLenti6-UbC-V5DEST. Young (PD< x +25) MRC5 were transduced with viral particles produced in Hek293FT cells using Virapower packaging mix as per the manufacturer's instructions (Life Technologies) and selected for 6 days with Blasticidin before utilisation.

Senescent cells. To generate replicatively senescent cells (MRC5 RepSen), MRC5 cells were cultured in complete medium at 20% oxygen, passaging before confluency, until PD < 0.1 per month. Stress induced senescent MRC5 (MRC5 IR) and IMR90 (IMR90 IR) were created by X-ray irradiation. 6300 cells/cm^2^ were seeded 24 h prior to 20 Gy irradiation and were used for experiments 7 days later. Oncogene-induced senescent cells (IMR90 OIS) were used on day 7 after induction of RAS overexpression in IMR-90ER:RAS by incubation with 100 nM 4-hydroxy-tamoxifen (4OHT).

For direct co-culture experiments, inducer senescent cells were plated at 5500 cells/cm^2^ density either on glass cover slips (No. 1.5) in 12 well plates or in 35 mm glass bottomed dishes (Willco) for live cell microscopy. Twenty four hours later, young bystander cells were plated in 1:1 ratio and co-cultures were maintained for 6 days. For treatment with chemical inhibitors: 1 μM Bay 11–7082, 10 nM Torin1, 1 μM UR-13756 (Bagley et al., 2010) or 100 U/ml catalase or DMSO as a control were added 24 h post seeding of bystanders and re-freshed every 3 days. TNFα was used at 10 ng/ml. Pre-treatment of inducer cells with TNFα was performed for 1 h before replacing with fresh medium and seeding bystander cells.

Treatment with siRNA was performed 24 h after seeding of bystander cells. Co-cultures were transfected using 5 nM Silencer select Pre-designed siRNA and Lipofectamine™ RNAiMAX (Thermo Fisher) in antibiotic-free medium. Forty-eight hours later, transfections were repeated and cells were fixed after 6 days in co-culture.

### 4.2 Detection of ROS

ROS production was assessed in cultured fibroblasts by staining trypsinised cells with 10 μM Dihydroethidium (DHE) and measuring fluorescence by flow cytometry as described previously (Dalle Pezze et al., 2014). For measurement of co-cultured cells, DHE fluorescence was measured by live cell microscopy to robustly separate young bystanders from senescent inducers (see Microscopy section below).

### 4.3 ELISA assays

Cells were incubated in fresh half normal volume of media for 40 h prior to collection. Samples were cleared by centrifugation for 10 min at 4 °C at 8000*g* and then concentrated 2 times by Cryospin. Protein concentration was measured using DC Protein assay (BioRad) according to the manufacturer's protocol. Concentrated conditioned media were assessed using the respective Sandwich ELISA DuoSet Development System kits (R&D Systems, Abingdon, UK). The kits were tested and suitably modified to be transferred onto a 384-well plate platform with the use of an EpMotion 5075 Liquid Handling robot. All colorimetric measurements were performed at 450 nm with wavelength correction at 550 nm on an Omega microplate reader (BMG Labtech, Aylesbury, UK) applying a four-parameter logistic curve fit to the respective standard curves. Data were normalized to total protein concentrations.

### 4.4 Microscopy

For end point analysis of mCherry fluorescence, cells were fixed for 10 min with 4% PFA and mounted using Prolong Diamond Antifade with DAPI (Thermo Fisher, UK), or were imaged by live cell microscopy.

For immunofluorescence, cells were fixed as above and incubated with rabbit anti-p65 (1:400, Cell Signalling) overnight at 4oC. For visualization of the staining, AlexaFluor 488 conjugated secondary antibody was used (Thermo Fisher).

Imaging was performed using a Zeiss Spinning Disk Confocal system, coupled with Quant-EM 512 SC camera (Photometrics) and Yokogawa CSU-X1 spinning disk head, with a 63 x (NA 1.4) objective, and a temperature and gas regulated incubator (Pecon). Image acquisition was via AxioVision software (Zeiss, UK) and image analysis (counts of mCherry DNA damage foci per nucleus, and RelA nuclear and cytosolic fluorescence intensities) was performed in ImageJ. To ensure accurate comparisons, all imaging within an experiment was performed using the same excitation intensities, exposure times and objectives. Also, to improve quantification, mean nuclear RelA fluorescence was normalised to the cells respective cytosolic RelA fluorescence staining. This removes any differences from staining variability from coverslip to coverslip, or any slight differences in focal plane or laser intensity from field to field. Population-level activation rates were determined by measuring the percentage of cells per field that had predominantly nuclear (i.e. nuclear: cytosolic fluorescence ratio of RelA staining was >1 per cell) RelA fluorescence.

For measurement of DHE fluorescence by live cell microscopy, MRC5 cells stably transduced with an EGFP-luc expression cassette (pSLIEW) were used as bystanders to identify them from the senescent parental line MRC5 inducers based upon nuclear EGFP fluorescence. Cells were plated as described above and stained with DHE as for flow cytometry measurements, then washed and replaced with Fluorobrite medium (Thermo Fisher) plus 10% FCS. Staining was performed in a serial manner to maintain the same incubation times per dish. Images were captured using a DMi8 widefield fluorescence microscope equipped with a 20 × 0.8NA objective, a Sola-SE LED light source (Lumencor) and a Flash4 sCMOS camera (Hamamatsu) using LASX software (Leica). EGFP, DHE and brightfield images were captured for each field using GFP and TX2 filter cubes (Leica) for the fluorescent channels representing EGFP and DHE respectively. Mean nuclear DHE fluorescence (from TX2 filterset) was determined using ImageJ for at least 100 cells from 3 different dishes per group, using the brightfield image to identify the nucleus. Intensities were background subtracted based upon mean+ 2xSD nuclear fluorescence intensity from TX2 images of young EGFP expressing cells without any DHE staining. In co-cultured dishes, bystander cells were identified by EGFP expression being present throughout the cell (using the GFP filterset image), and were identified by their nuclear EGFP fluorescence to allow discrimination from lipofuscin-based autofluorescence, visible in the cytosol of the senescent inducer cells.

### 4.5 Statistical analysis

All analysis was performed using SigmaPlot 12.5 software, using one-way ANOVA test (parametric data) or Kruskal-Wallis ANOVA (non-parametric). Post-hoc tests were performed pairwise within datasets using Dunn’s or Holm-Sidak method. Cut-off of significance was set at p < 0.05 (denoted as * in Figures). Two-way ANOVA test was used for assessment of the relationship between cell line background and mode of senescence or treatments.
