# Supplementary material for: The senescent bystander effect is caused by ROS-activated NF-κB signalling
Source: Mech Ageing Dev. 2018 Mar;170:30–6. doi: 10.1016/j.mad.2017.08.005 (PMC5861994; doi:10.1016/j.mad.2017.08.005)
Supplement: Supplementary file 1 [file mmc1.pdf]

A)

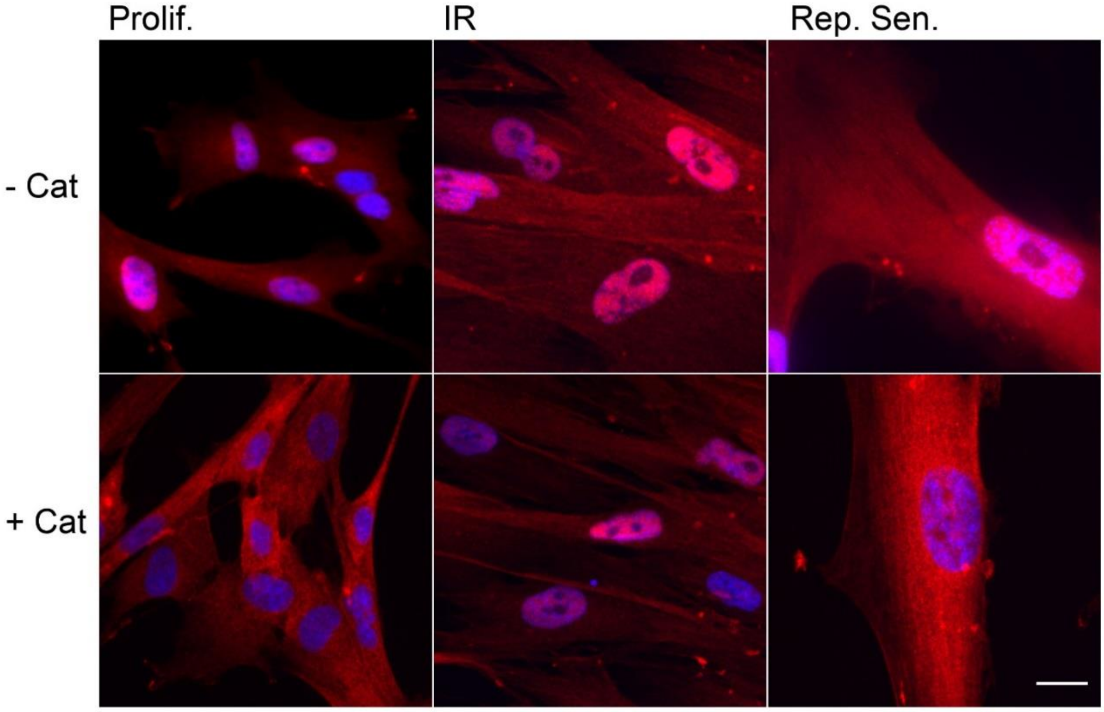

B)

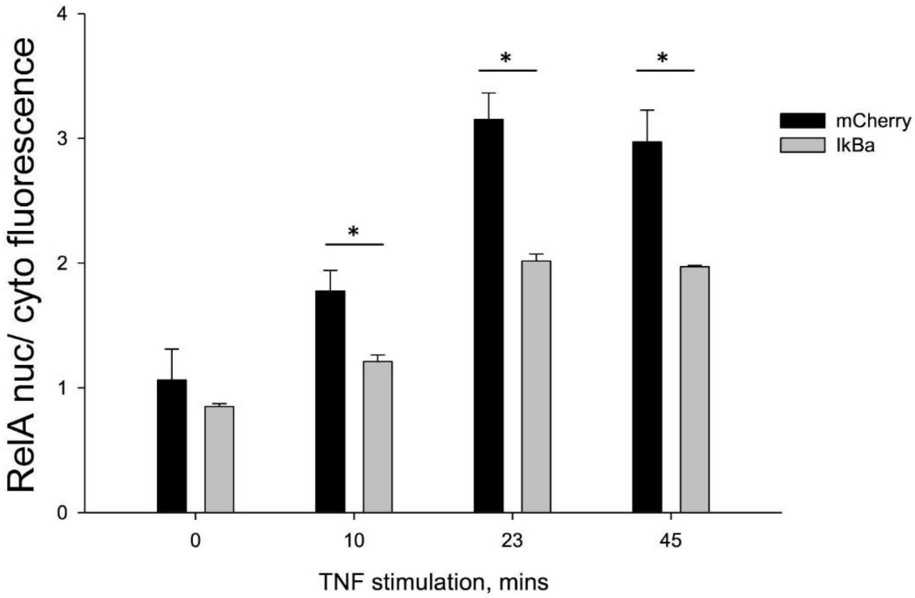

Figure S1. Nuclear/cytoplasmic RelA ratio as indicator of NF-κB activity.
A) Nuclear localisation of RelA increases in MRC5 senescence and is decreased by catalase treatment. Red: RelA immunofluorescence signal, blue: DAPI. Bar equals 20 μm.
B) Nuclear/cytoplasmic RelA ratio in cells stimulated with TNFα. MRC5 fibroblasts expressing ΔIκBα (IkBa) or mCherry were stimulated for the indicated times with 10 ng/ml TNFα and the ratio of nuclear /cytoplasmic RelA fluorescence was measured by immunofluorescence. n=3 with a minimum of 20 cells per timepoint analysed per replicate. Significantly lower activation levels of RelA were observed in ΔIκBα expressing cells at all time points post TNFα addition (1 way ANOVA with Holm Sidak post hoc test).

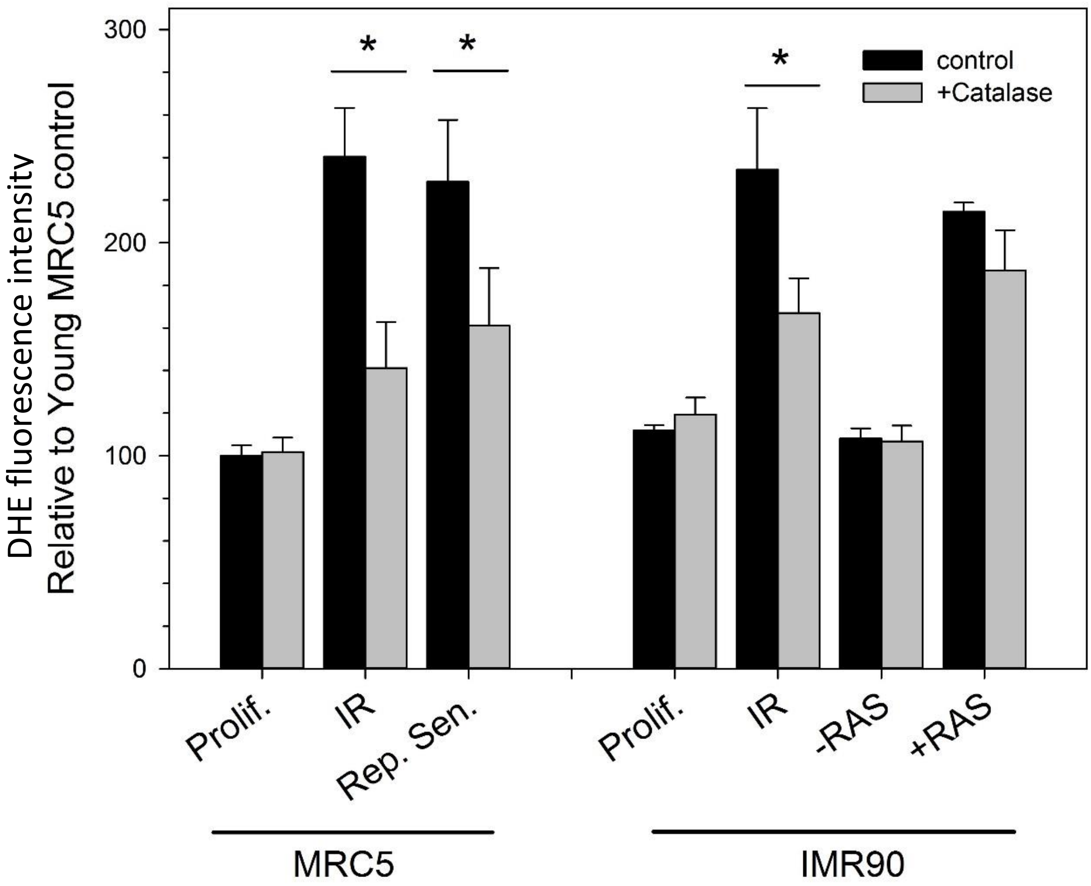

Figure S2. Catalase reduces ROS in senescence. Proliferating cells and cells in different senescence modes as indicated were treated for two days with catalase and cellular ROS levels were measured by DHE fluorescence intensity in flow cytometry. DHE fluorescence intensities are given relative to untreated young MRC5. n=3 independent replicates with 3 populations of cells (minimum 10,000 per population) measured per replicate. Significant differences are shown based on a 1 way ANOVA with Holm Sidak post hoc test.

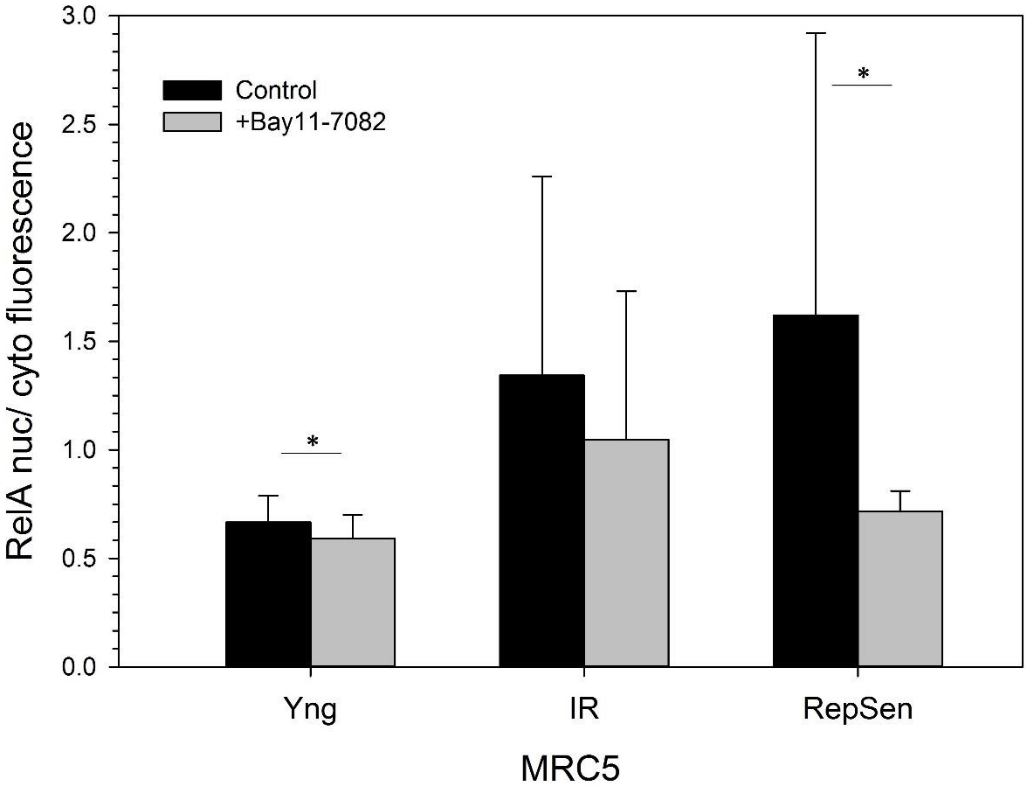

Figure S3: Treatment with the IKK inhibitor, Bay11-7082 for 2 days reduces NF-κB activation in senescence. Resting MRC5 cells were treated with Bay11-7082 (1 μM) or DMSO for 60 minutes and NF-κB activity was measured as the ratio of nuclear /cytoplasmic RelA fluorescence by immunofluorescence. Between 50 and 150 cells were analysed per treatment. Significantly lower NF-κB activation were observed in young and replicatively senescent cells respective to their controls (1 way ANOVA with Dunn's post hoc test).

A)

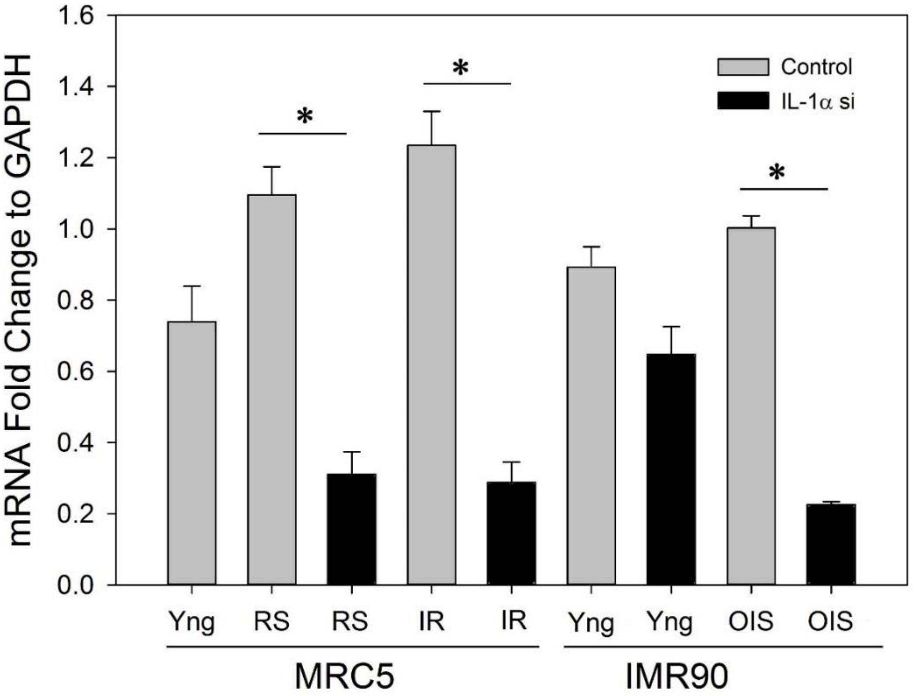

B)

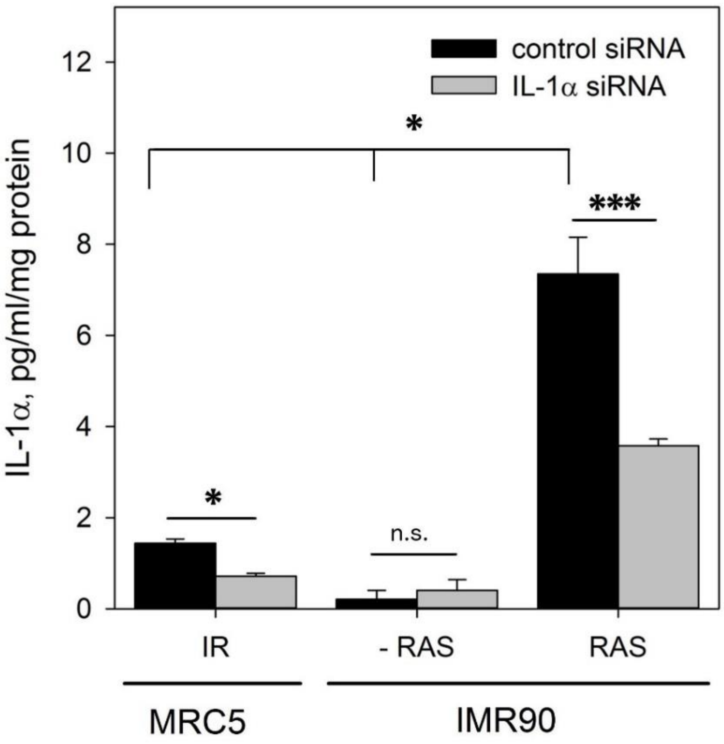

Figure S4: Effects of siRNA-mediated knock-down of IL-1α.

A) IL-1α is upregulated in multiple modes of senescence and is sensitive to siRNA knock-down. RT-PCR analysis of cells transfected with either scrambled or IL-1α siRNA 3 days prior to sample collection. siRNA-mediated knock-down of IL-1α significantly decreased the level of IL-1α

in all modes of senescence. Data plotted are mean +/- SD from 2 experiments with 3 replicates each. Significant differences shown based on a two-way ANOVA with Holm-Sidak post-hoc test.

B) Knockdown of IL-1α expression diminishes release of IL-1α from senescent cells. ELISA analysis of IL-1α levels in stress-induced senescence (MRC5) and oncogene induced senescence (IMR90) transfected with either scrambled or IL-1α siRNA 3 days prior to sample collection. Data plotted are mean +/- SD from 2 experiments with 3 replicates each. Significant differences shown based on a two-way ANOVA with Holm-Sidak post-hoc test.

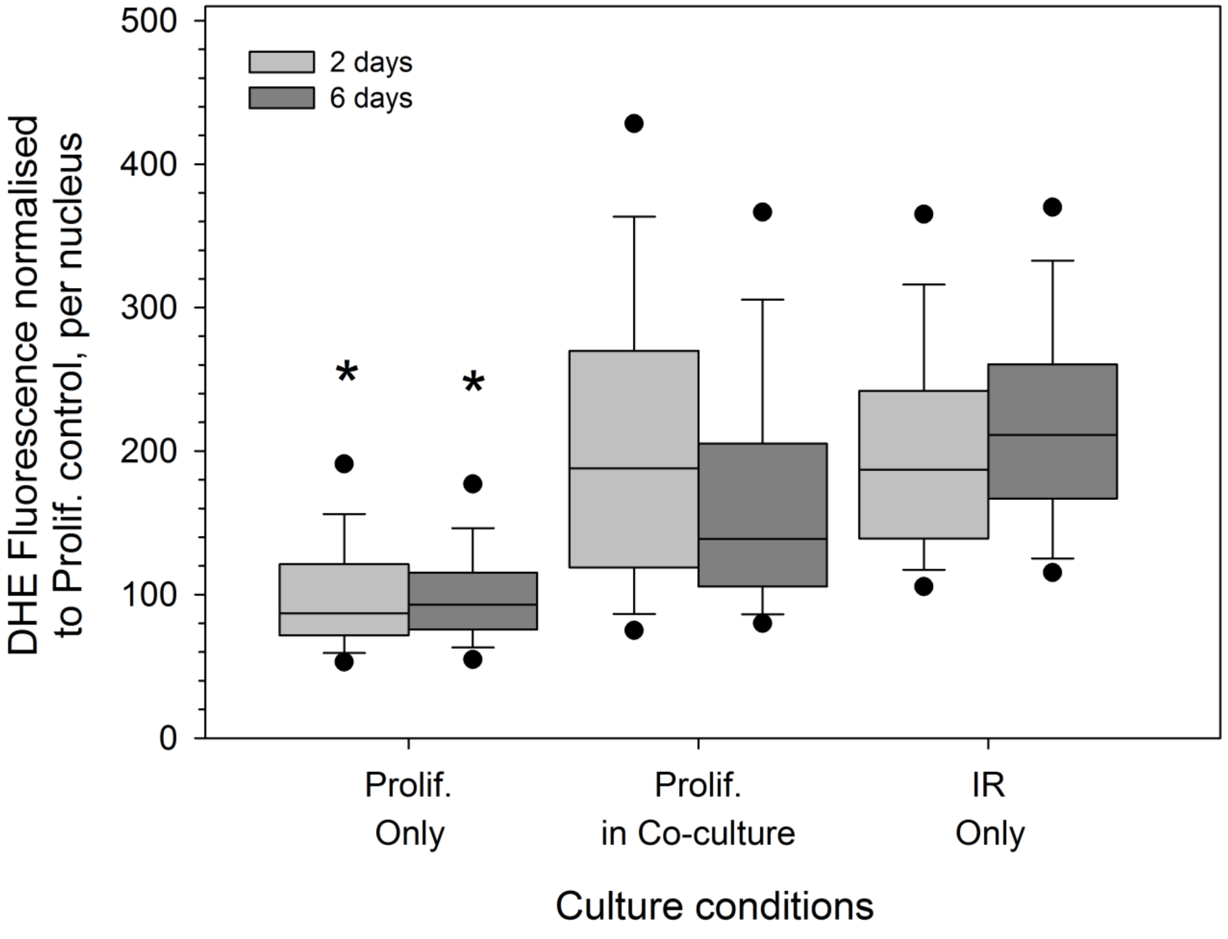

Figure S5: Co-culture with senescent cells enhances ROS in bystander cells. Senescent (IR) and GFP-expressing young MRC5 cells were maintained either separately or in co-culture for the indicated times and nuclear DHE fluorescence intensity was measured. Only bystander (GFP-expressing) cells were measured in co-culture. Senescent only cells and co-cultured proliferating (GFP positive) bystander cells exhibited significantly higher DHE fluorescence than proliferating cells alone at both time points investigated. Data plotted as box and whiskers with median frequencies and $5^{th}/95^{th}$ % outliers shown from 3 experiments and at least 100 cells per treatment/ timepoint. Significant differences shown based on a one-way ANOVA with Holm-Sidak post-hoc test for each time point.
